# Missed *Plasmodium falciparum* and *Plasmodium vivax* Mixed Infections in Ethiopia Threaten Malaria Elimination

**DOI:** 10.4269/ajtmh.21-0796

**Published:** 2021-11-30

**Authors:** Colleen M. Leonard, Hussein Mohammed, Mekonnen Tadesse, Jessica N. McCaffery, Doug Nace, Eric S. Halsey, Samuel Girma, Ashenafi Assefa, Jimee Hwang, Eric Rogier

**Affiliations:** ^1^ Malaria Branch, Division of Parasitic Diseases and Malaria, Centers for Disease Control and Prevention, Atlanta, Georgia;; ^2^ Ethiopia Public Health Institute, Addis Ababa, Ethiopia;; ^3^ ICAP at Columbia University, Addis Ababa, Ethiopia;; ^4^ U.S. President’s Malaria Initiative, Malaria Branch, Centers for Disease Control and Prevention, Atlanta, Georgia;; ^5^ U.S. President’s Malaria Initiative, USAID, Addis Ababa, Ethiopia;; ^6^ Infectious Disease Ecology and Epidemiology Lab, University of North Carolina at Chapel Hill, Chapel Hill, North Carolina

## Abstract

*Plasmodium falciparum* and *Plasmodium vivax* are co-endemic in Ethiopia. This study investigated whether mixed infections were missed by microscopy from a 2017 therapeutic efficacy study at two health facilities in Ethiopia. All patients (*N* = 304) were initially classified as having single-species *P. falciparum* (*n* = 148 samples) or *P. vivax* infections (*n* = 156). Dried blood spots were tested for *Plasmodium* antigens by bead-based multiplex assay for pan-*Plasmodium* aldolase, pan-*Plasmodium* lactate dehydrogenase, *P. vivax* lactate dehydrogenase, and histidine-rich protein 2. Of 304 blood samples, 13 (4.3%) contained both *P. falciparum* and *P. vivax* antigens and were analyzed by polymerase chain reaction for species-specific DNA. Of these 13 samples, five were confirmed by polymerase chain reaction for *P. falciparum*/*P. vivax* co-infection. One sample, initially classified as *P. vivax* by microscopy, was found to only have *Plasmodium ovale* DNA. *Plasmodium falciparum/P. vivax* mixed infections can be missed by microscopy even in the context of a therapeutic efficacy study with multiple trained readers.

Ethiopia is one of the few African countries where both *Plasmodium falciparum* and *Plasmodium vivax* species are co-endemic at substantial proportions, and are accounted for in malaria diagnostic and treatment guidelines.^1^ *Plasmodium falciparum* accounts for ≈60% of all cases; *P. vivax* accounts for most of the remaining cases in Ethiopia.^2^ To prevent morbidity and mortality resulting from malaria, the Ethiopian Ministry of Health aims to achieve malaria elimination by 2030.^3^ To facilitate malaria elimination and prevent severe disease, accurate diagnosis and effective treatment of all malaria cases are essential. The Ministry of Health requires malaria diagnosis by either microscopy or rapid diagnostic test (RDT).^3^ Microscopy allows for laboratory technicians to distinguish between different malaria species; however, the accuracy is limited by the sample’s parasite density and the technician’s expertise.^4^ The detection limit of microscopy and RDTs is generally considered to be ≈100 parasites/µL.^5^

Persons may become infected with multiple malaria parasites simultaneously.^6^ However, mixed infections are likely underreported because they are often difficult to detect by microscopy and unable to be detected by many RDTs.^4^^,^^7^ Ayalew et al.^4^ reported that only about 45% of microscopists from Ethiopian hospitals and health centers accurately identified a *P. falciparum*/*P. vivax* mixed infection. RDTs detecting histidine-rich protein 2 (HRP2) and/or pan-*Plasmodium* lactate dehydrogenase antigens are unable to distinguish between a *P. falciparum* and *P. falciparum*/*P. vivax* mixed infection.^7^ *Plasmodium falciparum*/*P. vivax* combination-test RDTs are available that can detect HRP2 and *P. vivax*-specific LDH (PvLDH) and therefore can identify *P. falciparum*/*P. vivax* mixed infections; however, they cannot detect *P. malariae* or *P. ovale*. Accurate diagnosis of multiple malaria species is important for effective treatment and public health surveillance in a region.

Tests more sensitive than microscopy and RDTs are available for detecting *Plasmodium* infection, including polymerase chain reaction (PCR) and bead-based multiplex antigen detection assays, but these methods are limited to laboratory settings because they require specialized equipment and skilled technicians.^8^ The bead-based antigen assay simultaneously detects multiple *Plasmodium* antigens from a blood sample and provides sensitivity comparable to PCR.^9^ To identify malaria mixed infections, samples from a 2017 therapeutic efficacy study (TES) in Ethiopia were analyzed using a bead-based multiplex antigen detection assay and PCR to assess samples initially classified as single-species *P. falciparum* or *P. vivax* infections by microscopy. Persons presenting with symptoms of malaria and diagnosed via microscopy with a parasite density of 500 to 100,000 asexual parasites/μL of blood for *P. falciparum* or more than 250 asexual parasites/μL of blood for *P. vivax* were eligible for the study. Only single-species *P. falciparum* or *P. vivax* infections were eligible for inclusion. RDTs (*P. falciparum*/pan-*Plasmodium*) were also conducted at the time of enrollment, but the results did not affect enrollment procedures. Treatment of malaria was provided according to the study protocol with artemether–lumefantrine or dihydroartemisinin–piperaquine for *P. falciparum* infection and chloroquine or dihydroartemisinin–piperaquine for *P. vivax* infection. Individuals presenting with *P. vivax* and testing normal for glucose-6-phosphate dehydrogenase were offered primaquine radical cure at the end of the 42-day follow-up period. The TES protocol was approved by the Ethiopian Public Health Institute, the National Ethical Committee (3.10/171/2016) and the Food, Medicine and Health Care Administration and Control Authority in Ethiopia (02/6/9-1/81). In addition, the study was reviewed by Columbia University (AAAQ9414) and the CDC Human Subjects office (no. 6892.0) and was conducted consistent with applicable U.S. federal law and CDC policy.

Two study sites were included in the TES: Pawe (Benishangul Gumuz region) and Arba Minch (Southern Nations, Nationalities, and Peoples’ region). For samples with dried blood spots (DBS) available for laboratory evaluation (*N* = 304), microscopy diagnosis identified 148 with single-species *P. falciparum* (136 from Pawe and 12 from Arba Minch) and 156 samples with single-species *P. vivax* infections (60 from Pawe and 96 from Arba Minch). Slides were read by a study site microscopist and then re-examined blinded by a WHO-certified level-1 and level-2 microscopist.

All 304 DBS samples were analyzed by bead-based multiplex antigen assay for pan-*Plasmodium* aldolase, pan-*Plasmodium* lactate dehydrogenase, PvLDH, and HRP2. The PvLDH and HRP2 antigens are species-specific markers for *P. vivax* and *P. falciparum*, respectively. From the multiplex antigen screening of all samples, the majority showed species-specific antigen concordance with the participants’ microscopy results: 145/148 *P. falciparum* infections (98.0%) and 153/156 *P. vivax* infections (98.1%). However, 15 of the 304 samples (4.9%) showed the presence of both species-specific antigens, nine of which were identified previously as *P. falciparum* and six as *P. vivax* by microscopy. A scatterplot for these two species-specific antigen levels and designation of the double positives is shown in Figure 1.

**Figure 1.**
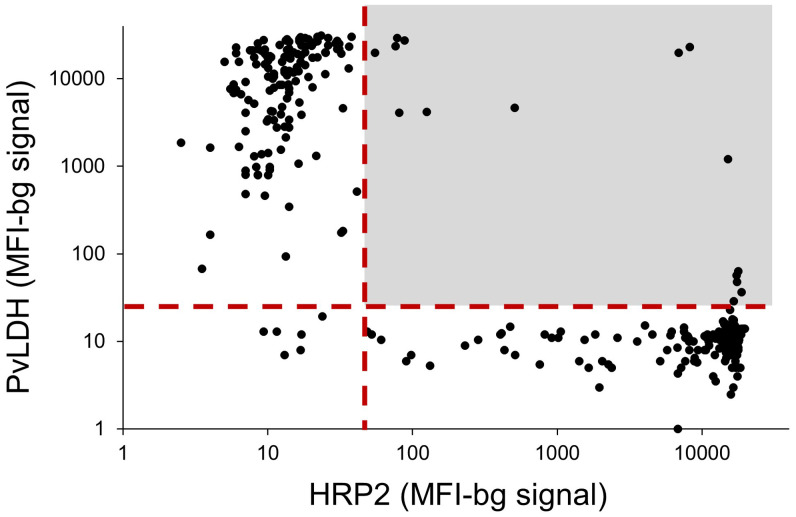
Scatterplot of *Plasmodium falciparum* histidine-rich protein 2 (HRP2) assay signal compared with *Plasmodium vivax* lactate dehydrogenase (PvLDH) assay signal for 304 dried blood spot samples. The vertical dashed red line designates the threshold for the antigen positivity signal for the HRP2 antigen; the horizontal dashed red line designates the threshold for the antigen positivity signal for PvLDH. The lightly shaded region denotes values where dried blood spot samples were positive for both species-specific antigens. MFI-bg = median fluorescence intensity minus background fluorescence. This figure appears in color at www.ajtmh.org.

Of the 15 blood samples containing both species-specific antigens,13 had enough blood remaining for DNA extraction for PCR assays to investigate potential mixed-species infections (Table 1). One additional sample identified as containing *P. vivax* by microscopy but PvLDH negative and HRP2 positive was also subjected to PCR testing. For these 14 samples, DNA was extracted from a single 6-mm DBS punch using the Qiagen DNA Mini Kit (QIAGEN, Germantown, MD), and photo-induced electron transfer–PCR with species-specific primers was performed as described previously.^10^ Of the seven microscopy-positive *P. falciparum* samples with DBS positive for PvLDH (and HRP2), all (100%) were also found to have *P. vivax* DNA. Of the six microscopy-positive *P. vivax* samples with DBS positive for HRP2 (and PvLDH), one (16.7%) was found to contain *P. falciparum* and *P. vivax* DNA. The sample initially identified with *P. vivax* infection by microscopy but PvLDH negative was found to contain *P. ovale* DNA, but neither *P. falciparum* nor *P. vivax* DNA. *Plasmodium ovale* is likely underdiagnosed in Ethiopia because the estimated seroprevalence for *P. ovale* is 3.1%.^11^ From the combination of antigen and DNA data, of the 304 clinical samples assessed, five (1.6%) were found to be *P. falciparum*/*P. vivax* mixed infections and one (0.3%) was a *P. ovale* infection. Most of the mixed infections (80%) were identified as *P. falciparum* by microscopy. Three samples identified as *P. falciparum* by microscopy and RDT, but noted to contain *P. vivax* DNA only, were likely *P. vivax* mono-infections in the setting of a recently cleared *P. falciparum* infection. *Plasmodium falciparum* diagnosis by microscopy was less accurate compared with *P. vivax* diagnosis as three of seven *P. falciparum* diagnoses were missing *P. falciparum* DNA compared with one of seven *P. vivax* diagnoses missing *P. vivax* DNA. Table 1 shows the assay results, study sites, demographics, and clinical information for these 14 samples.

**Table 1:** Results from microscopy, antigen detection, and polymerase chain reaction assays for samples selected with suspicion of *Plasmodium falciparum*/*Plasmodium vivax* mixed infections

Sample no.	Microscopy diagnosis	Study site	Treatment provided	Parasite density by microscopy (parasites/μL blood)	RDT	Gender	Age (y)	Hgb (g/dL)	HRP2 (pg/mL)	PvLDH (pg/mL)	PET-PCR Pf Ct*	PET-PCR Pv Ct
1	Pf	Pawe	AL	26,820	Pf	M	11	10.8	1,881,181	871	26.9	36.6
2	Pf	Pawe	AL	17,480	Pf	M	17	10.1	1,118,668	655	28.2	39.9
3	Pf	Pawe	AL	6,834	Pf	F	16	15.8	1,162	54,941	–	29.4
4	Pf	Pawe	AL	18,246	Pf	M	17	14.0	106,889	14,333	24.9	35.8
5	Pf	Pawe	AL	78,238	Pf	F	11	12.4	913,114	778	23.3	39.3
6	Pf	Pawe	AL	8,142	Pf	M	20	12.9	166	47,572	–	30.0
7	Pf	Arba Minch	AL	2,480	Pf	F	19	13.7	28,354	25,353	44.4	27.7
8	Pv	Pawe	CQ	22,246	Pv	F	5	11.4	113	824,001	39.0	28.3
9	Pv	Pawe	CQ	16,038	Pv	M	15	14.8	160	27,616	41.4	27.4
10†	Pv	Pawe	CQ	5,200	Neg†	M	15	14.1	100	0.0†	–	–
11	Pv	Pawe	CQ	9,595	Pv	F	9	12.4	260	48,688	–	28.4
12	Pv	Arba Minch	CQ	48,320	Pv	M	20	16.1	156	24,225	–	27.7
13	Pv	Arba Minch	DP	27,200	Pv	M	23	12.8	22,272	830,205	–	27.5
4	Pv	Arba Minch	CQ	11,720	Pv	M	3	12.6	181	26,483	44.2	26.7

AL = artemether lumefantrine; CQ = chloroquine; Ct = cycle threshold value; DP = dihydroartemisinin–piperaquine; F = female; Hgb = hemoglobin; HRP2 = histidine-rich protein 2; M = male; PET-PCR = photo-induced electron transfer polymerase chain reaction; Pf = *Plasmodium falciparum*; Pv = *Plasmodium vivax*; PvLDH = *P. vivax* lactate dehydrogenase; RDT = rapid diagnostic test.

* A Ct value less than 40.0 is considered DNA positive; a negative sign indicates no amplification

† Sample had a negative assay signal for PvLDH; found to contain *Plasmodium ovale* DNA.

Seven samples identified initially as *P. falciparum* infections by microscopy were later found to contain PvLDH antigens and *P. vivax* DNA. Because LDH clears from circulation quickly after parasite clearance,^12^ the concomitant PvLDH antigenemia and *P. vivax* DNA are not surprising. However, of the six samples with potential *P. falciparum/P. vivax* mixed infection based on a *P. vivax* microscopy result and the presence of HRP2 antigens, only one contained *P. falciparum* DNA. This could represent a newly acquired *P. falciparum* infection without a high-enough parasitemia to be identified easily by microscopy. In addition, the one sample with *P. ovale* DNA also contained HRP2 antigens. Unlike DNA, HRP2 can persist in the circulation for months after a *P. falciparum* infection and may be detected coincidentally during an ongoing or recent *P. vivax* (or *P. ovale*) infection.^13^^,^^14^ In some cases, there may be sufficient antigen levels in a DBS sample to be detected, but not enough DNA to be detected by PCR.^15^ The bead-based antigen assay provides a more efficient method to screen samples for potential mixed infection compared with testing all samples using PCR, which is more expensive and time-consuming.

In different areas of Ethiopia, the proportion of *P. falciparum/P. vivax* mixed infections is estimated to be between 0.5% to 5.0% of malaria cases.^16^^,^^17^ The value reported here (1.6%) is likely an underestimate because mixed infections were excluded from the TES. Missing mixed infections by microscopy is consistent with findings in other settings.^18^^,^^19^ Mixed infections may be missed by microscopic examination of blood films with low parasite density of one species or difficulty differentiating the lower density species from the dominant species.^20^ In either case, refresher training with a focus on identifying *P. falciparum/P. vivax* mixed infections would be beneficial. However, often, a highly sensitive method, such as PCR, is needed to uncover mixed infections.^19^

In areas where *P. falciparum* and *P. vivax* are endemic, uncovering *P. falciparum*/*P. vivax* mixed infections is important for the success of malaria control programs, elimination efforts, and effective clinical treatment. The recommended treatment of *P. falciparum*/*P. vivax* mixed infections in Ethiopia is artemether–lumefantrine plus radical cure with primaquine (0.25 mg/kg for 14 days).^3^ For *P. falciparum,* the recommended treatment is artemether–lumefantrine and single-dose primaquine (0.25 mg/kg); whereas for *P. vivax*, chloroquine and radical cure with primaquine (0.25 mg/kg for 14 days) is recommended.^1^ If a mixed infection is misdiagnosed as a *P. falciparum* or *P. vivax* mono-infection, then the given treatment would be inadequate. Although microscopy is the gold standard, missing mixed infections may lead to treatments that would fail to clear the presenting blood-stage infection or would miss clearing hypnozoites, either of which could impede malaria elimination efforts.

## References

[1] Ethiopia Ministry of Health , 2017. *National Malaria Guidelines*, 4th edition. Addis Ababa, Ethiopia: Ethiopian Federal Ministry of Health.

[2] Taffese HS Hemming-Schroeder E Koepfli C Tesfaye G Lee MC Kazura J Yan GY Zhou GF , 2018. Malaria epidemiology and interventions in Ethiopia from 2001 to 2016. Infect Dis Poverty 7: 1--9. 30392470 10.1186/s40249-018-0487-3 PMC6217769

[3] Ethiopian Ministry of Health , 2017. National Malaria Elimination Road Map. Addis Ababa, Ethiopia: Federal Ministry of Health.

[4] Ayalew F Tilahun B Taye B , 2014. Performance evaluation of laboratory professionals on malaria microscopy in Hawassa Town, southern Ethiopia. BMC Res Notes 7: 1--8. 25422030 10.1186/1756-0500-7-839 PMC4255633

[5] World Health Organization , 2013. *WHO Evidence Review Group on Malaria Diagnosis in Low Transmission Settings*. Available at: https://www.who.int/malaria/mpac/mpac_mar2014_diagnosis_low_transmission_settings_report.pdf. Accessed November 15, 2021.

[6] Mayxay M Pukrittayakamee S Newton PN White NJ , 2004. Mixed-species malaria infections in humans. Trends Parasitol 20: 233–240. 15105024 10.1016/j.pt.2004.03.006

[7] Moody A , 2002. Rapid diagnostic tests for malaria parasites. Clin Microbiol Rev 15: 66–78. 11781267 10.1128/CMR.15.1.66-78.2002 PMC118060

[8] World Health Organization , 2017. A Framework for Malaria Elimination. Geneva, Switzerland: WHO.

[9] Rogier E 2017. Bead-based immunoassay allows sub-picogram detection of histidine-rich protein 2 from *Plasmodium falciparum* and estimates reliability of malaria rapid diagnostic tests. PLoS One 12: e0172139. 28192523 10.1371/journal.pone.0172139 PMC5305216

[10] Escobar DF Lucchi NW Abdallah R Valenzuela MT Udhayakumar V Jercic MI Chenet SM , 2020. Molecular and epidemiological characterization of imported malaria cases in Chile. Malar J 19: 1--9. 32792011 10.1186/s12936-020-03353-y PMC7427082

[11] Assefa A 2019. Multiplex serology demonstrates cumulative prevalence and spatial distribution of malaria in Ethiopia. Malar J 18: 1--14. 31331340 10.1186/s12936-019-2874-z PMC6647069

[12] Plucinski MM McElroy PD Dimbu PR Fortes F Nace D Halsey ES Rogier E , 2019. Clearance dynamics of lactate dehydrogenase and aldolase following antimalarial treatment for *Plasmodium falciparum* infection. Parasit Vectors 12: 1--6. 31182154 10.1186/s13071-019-3549-x PMC6558726

[13] Plucinski MM 2018. Posttreatment HRP2 clearance in patients with uncomplicated *Plasmodium falciparum* malaria. J Infect Dis 217: 685–692. 29220497 10.1093/infdis/jix622 PMC11023016

[14] Commons RJ Simpson JA Thriemer K Hossain MS Douglas NM Humphreys GS Sibley CH Guerin PJ Price RN , 2019. Risk of *Plasmodium vivax* parasitaemia after *Plasmodium falciparum* infection: a systematic review and meta-analysis. Lancet Infect Dis 19: 91–101. 30587297 10.1016/S1473-3099(18)30596-6 PMC6300482

[15] Plucinski MM 2019. Screening for Pfhrp2/3-deleted *Plasmodium falciparum*, non-falciparum, and low-density malaria infections by a multiplex antigen assay. J Infect Dis 219: 437–447. 30202972 10.1093/infdis/jiy525 PMC6325347

[16] Argaw MD Woldegiorgis AG Abate DT Abebe ME , 2016. Improved malaria case management in formal private sector through public private partnership in Ethiopia: retrospective descriptive study. Malar J 15: 1--11. 27401095 10.1186/s12936-016-1402-7 PMC4940756

[17] Tadesse F Fogarty AW Deressa W , 2017. Prevalence and associated risk factors of malaria among adults in East Shewa Zone of Oromia Regional State, Ethiopia: a cross-sectional study. BMC Public Health 18: 1--8. 28716009 10.1186/s12889-017-4577-0 PMC5513396

[18] Fançony C Gamboa D Sebastião Y Hallett R Sutherland C Sousa-Figueiredo JC Nery SV , 2012. Various pfcrt and pfmdr1 genotypes of *Plasmodium falciparum* cocirculate with *P. malariae*, *P. ovale* spp., and *P. vivax* in northern Angola. Antimicrob Agents Chemother 56: 5271–5277. 22850519 10.1128/AAC.00559-12 PMC3457352

[19] Snounou G Viriyakosol S Jarra W Thaithong S Brown KN , 1993. Identification of the four human malaria parasite species in field samples by the polymerase chain reaction and detection of a high prevalence of mixed infections. Mol Biochem Parasitol 58: 283–292. 8479452 10.1016/0166-6851(93)90050-8

[20] Richie TL , 1988. Interactions between malaria parasites infecting the same vertebrate host. Parasitology 96: 607–639. 3043327 10.1017/s0031182000080227

